# Homoharringtonine is highly effective against SARS-CoV-2: a potential first-line defense in future coronavirus epidemics

**DOI:** 10.1093/nsr/nwae382

**Published:** 2024-10-26

**Authors:** Hai-Jun Wen, Hua-Juan Ma, Gong-Xun Zhong, Ao Ding, Xi Wang, Xiao-Ju Lai, Chang-Hao Shi, Miles Tracy, Si-Jin Wu, Jia-Sheng Li, Ge Zhang, Yong-Bo Yao, Wei Chen, Xue-Yu Liu, Zhi-Chao Xu, Xue Cao, Wen-Bin He, Jing Feng, Guo-Dong Wang, Feng-Liang Liu, Wei-Shun Zeng, Guang-An Lu, Jia-Yu Guo, Yong-Tang Zheng, Hui Zeng, Xiong-Lei He, Fu-Jie Zhang, Zhi-Hong Hu, Xue-Mei Lyu, Hualan Chen, Xin-Xing Lei, Jian Li, Man-Li Wang, Qi-Chun Cai, Chung-I Wu

**Affiliations:** State Key Laboratory of Biocontrol, School of Life Sciences, Sun Yat-sen University, Guangzhou 510275, China; Cancer Center, Clifford Hospital, Jinan University, Guangzhou 511495, China; State Key Laboratory of Veterinary Biotechnology, Harbin Veterinary Researched Institute, Chinese Academy of Agricultural Sciences, Harbin 150069, China; State Key Laboratory of Biocontrol, School of Life Sciences, Sun Yat-sen University, Guangzhou 510275, China; State Key Laboratory of Virology, Wuhan Institute of Virology, Chinese Academy of Sciences, Wuhan 430071, China; State Key Laboratory of Biocontrol, School of Life Sciences, Sun Yat-sen University, Guangzhou 510275, China; State Key Laboratory of Biocontrol, School of Life Sciences, Sun Yat-sen University, Guangzhou 510275, China; State Key Laboratory of Biocontrol, School of Life Sciences, Sun Yat-sen University, Guangzhou 510275, China; Academy of Pharmacy, Xi'an Jiaotong-Liverpool University, Suzhou 215123, China; State Key Laboratory of Biocontrol, School of Life Sciences, Sun Yat-sen University, Guangzhou 510275, China; Cancer Center, Clifford Hospital, Jinan University, Guangzhou 511495, China; Cancer Center, Clifford Hospital, Jinan University, Guangzhou 511495, China; Cancer Center, Clifford Hospital, Jinan University, Guangzhou 511495, China; State Key Laboratory of Biocontrol, School of Life Sciences, Sun Yat-sen University, Guangzhou 510275, China; State Key Laboratory of Biocontrol, School of Life Sciences, Sun Yat-sen University, Guangzhou 510275, China; Department of Laboratory Animal Science, Kunming Medical University, Kunming 650500, China; State Key Laboratory of Genetic Resources and Evolution, Kunming Institute of Zoology, Chinese Academy of Science, Kunming 650223, China; State Key Laboratory of Genetic Resources and Evolution, Kunming Institute of Zoology, Chinese Academy of Science, Kunming 650223, China; State Key Laboratory of Genetic Resources and Evolution, Kunming Institute of Zoology, Chinese Academy of Science, Kunming 650223, China; Key Laboratory of Animal Models and Human Disease Mechanisms of Chinese Academy of Sciences / Key Laboratory of Bioactive Peptides of Yunnan Province, Kunming Institute of Zoology, Chinese Academy of Sciences, Kunming 650223, China; Kunming National High-level Bio-safety Research Center for Non-human Primates, Center for Biosafety Mega-Science, Kunming Institute of Zoology, Chinese Academy of Sciences, Kunming 650107, China; State Key Laboratory of Biocontrol, School of Life Sciences, Sun Yat-sen University, Guangzhou 510275, China; State Key Laboratory of Biocontrol, School of Life Sciences, Sun Yat-sen University, Guangzhou 510275, China; State Key Laboratory of Biocontrol, School of Life Sciences, Sun Yat-sen University, Guangzhou 510275, China; Key Laboratory of Animal Models and Human Disease Mechanisms of Chinese Academy of Sciences / Key Laboratory of Bioactive Peptides of Yunnan Province, Kunming Institute of Zoology, Chinese Academy of Sciences, Kunming 650223, China; Kunming National High-level Bio-safety Research Center for Non-human Primates, Center for Biosafety Mega-Science, Kunming Institute of Zoology, Chinese Academy of Sciences, Kunming 650107, China; Beijing Ditan Hospital, Capital Medical University, Beijing 100102, China; State Key Laboratory of Biocontrol, School of Life Sciences, Sun Yat-sen University, Guangzhou 510275, China; Beijing Ditan Hospital, Capital Medical University, Beijing 100102, China; State Key Laboratory of Virology, Wuhan Institute of Virology, Chinese Academy of Sciences, Wuhan 430071, China; State Key Laboratory of Genetic Resources and Evolution, Kunming Institute of Zoology, Chinese Academy of Science, Kunming 650223, China; State Key Laboratory of Veterinary Biotechnology, Harbin Veterinary Researched Institute, Chinese Academy of Agricultural Sciences, Harbin 150069, China; Department of Laboratory Medicine, Shenzhen People's Hospital (The First Affiliated Hospital, Southern University of Science and Technology; The Second Clinical Medical College, Jinan University) Shenzhen 518020, China; Hangzhou MinSheng Pharm. Group, Hangzhou 310005, China; State Key Laboratory of Virology, Wuhan Institute of Virology, Chinese Academy of Sciences, Wuhan 430071, China; Cancer Center, Clifford Hospital, Jinan University, Guangzhou 511495, China; State Key Laboratory of Biocontrol, School of Life Sciences, Sun Yat-sen University, Guangzhou 510275, China; Southern Marine Science and Engineering Guangdong Laboratory (Zhuhai), Zhuhai 519082, China

**Keywords:** homoharringtonine, coronavirus, SARS-CoV-2

## Abstract

As COVID-19 is the third coronavirus epidemic in this century, it would be desirable to have a treatment scheme in anticipation of a fourth one. We here present a scheme that could clear SARS-CoV-2 in 2–4 days post-infection from the upper respiratory tract (URT), where the virions are initially concentrated. The scheme, applicable on a large scale by nasal spray, is based on homoharringtonine (HHT), which has been approved for treating other diseases. HHT blocked protein elongation and repressed *in vitro* replication of all four coronaviruses (including SARS-CoV-2) that were tested at the nano-molar concentration, demonstrating its potential for broad effectiveness against coronaviruses. In animal models, HHT cleared SARS-CoV-2 in all treated mice within 3 days by daily nasal dripping of a small dose (40 μg). In December 2022, HHT was administered to 26 cancer patients by nebulization at 1 mg/day. On average, the viral load in the URT was reduced by three-quarters 6 hours after the nebulization. In the wavelet of May 2023, 11 patients without other medical conditions were administered HHT by repeated liquid nasal spray at the low total daily dose of 0.2 mg. Ten of the 11 patients were cleared of the virus in 2–4 days. In comparison, in large-cohort studies of participants in China during the same wave, most patients need 7–9 days to turn negative. No adverse effects were detected in any patient in the two clinical trials. A short review of drugs approved for treating COVID-19 shows the many advantages of HHT. With continual development, it could become a first-line defense at the onset of future coronavirus epidemics.

## INTRODUCTION

In the early stages of COVID-19, we proposed a treatment scheme to clear the virus quickly, and on a large scale, from the upper respiratory track soon after infection [1,2]. The scheme is based on homoharringtonine (HHT), which is a plant compound that has been approved to treat other human diseases. The proposal was in response to the exigency of 2020 when the epidemics were spreading globally. It was a call for further development and possible implementation by any qualified facility. With substantial efforts, to be described in this study, HHT was then applied to COVID-19 patients at the peak of the COVID-19 epidemic in China in December 2022.

Since the WHO declared the end of COVID-19 in October 2023, the need for medical measures

against SARS-CoV-2 has waned [3,4]. Indeed, the dominant strain in the entire period of 2022–2024 has been Omicron—a ‘well-behaved’ pathogen with weak virulence but strong transmission. While COVID-19 seems unlikely to resurge, there is a caveat: after 2 years of circulation in human populations, the latest Omicron strains such as JN-1 vis-à-vis its relatives appear quite different and comparable to strains of different families [5]. Therefore, it remains somewhat uncertain whether COVID-19 will resurge or not, while it keeps flaring up sporadically at different times and localities [6–8].

In the long run, other viruses could start new epidemics. After all, COVID-19 is the third epidemic to have been caused by a coronavirus in the last 20 years. The global human conditions that underlie COVID-19 such as dense habitation, intense social interactions and rapid human movements have become increasingly conducive for new epidemics [9]. For public health, the task is to develop a broadly implementable plan to repress new coronaviruses at the very beginning of new epidemics. As previous coronaviruses are concentrated in the nasal, pharyngeal and oral cavities [10] in new infections, clearance of the viral load in the upper respiratory tract (URT) immediately after any sign of infection is crucial. Most importantly, this first-line defense should benefit the population by severing the transmission chain.

In a nascent epidemic, there will not be new drugs or vaccines. Social distancing is the last resort but, under a barely noticeable threat, it is impractical. Furthermore, after medical means are developed, the exigency of the epidemics may not permit the luxury of comprehensively testing their efficacies. In retrospect, with the benefit of time, drugs that were developed for COVID-19 are now being reported as being not much better than placebos [11–13]. Promising drugs in the earlier time of the pandemic, such as remdesivir, molnupiravir and PAXLOVID, are no exceptions. In the ‘Discussion’, we will provide a more extensive review of extant COVID-19 medications.

As the severance of the transmission chain is a population-level task, two additional requirements are critical. First, it has to be easily applicable on a large scale, both economically and logistically. Second, the scheme should be effective against new variants of the same strain. We now show that, by targeting the protein translation apparatus, HHT is insensitive to variants of SARS-CoV-2. Its effect against the pathogen, but not the host, is due to the manner of protein translation that is peculiar to the coronavirus, as will be shown here. The main objective of this study is to urge the continual development of HHT as a first-line defense in anticipation of future coronavirus epidemics.

## RESULTS

Among all known RNA viruses, coronaviruses have the largest genomes (26.4–31.7 kb) and use two-thirds of their genomes to make a super-peptide that comprises ∼16 non-structural proteins (NSPs). The super-peptide is synthesized in concatenation and later proteolysed into its component proteins [14]. In SARS-CoV-2, as well as its relatives, the super-protein is ∼8000 amino acids in size (molecular weight > 700 kDa). In contrast, peptides of its host (e.g. human) cell have a median length of ∼400 amino acids (molecular weight ∼38 kDa) [15]. The few large genes (∼5000 amino acids long) in the human genome are often highly tissue-specific with a relatively long half-life [16]. In summary, the viral super-protein stands out in comparison with the proteins of the host and thus may be vulnerable to suppression [1].

### The specific mechanism of suppression by HHT

We hypothesized that the peculiar way of making a super-protein for later proteolysis made coronaviruses vulnerable [1]. First, a drug that blocks the translation can be highly effective against the virus, as one blockage can abrogate all 16 NSPs. Second, if a drug works to block the elongation, a low dose may block the translation of >5000 amino acids but spare the smaller peptides. Third, since coronaviruses translate this super-peptide with an unusual step of frameshift about halfway into the process [17], any drug that interferes with translation may further disrupt the making of viral proteins.

HHT—or omacetaxine mepesuccinate in its semi-synthetic form—is a cytotoxic plant alkaloid that is extracted from *Cephalotaxus* species and is likely to be the strongest inhibitor of protein translation that has been approved for clinical use [18]. As will be presented later, EC_50_ (efficacy against SARS-CoV-2) is >1000-fold lower than CC_50_ (cell toxicity). HHT has been commonly used to treat leukemia patients since the 1970s. It is the first agent to have been approved by the FDA (USA, in 2012) for targeting the mRNA translation process [19]. In particular, HHT is effective against leukemic cells with hyperactive oncogenes [20]. Another drug (plitidepsin) also targets the translation machinery of a cell for the treatment of COVID-19 but it has a modest selectivity index (SI) of 40.4 [21].

**Figure 1.**
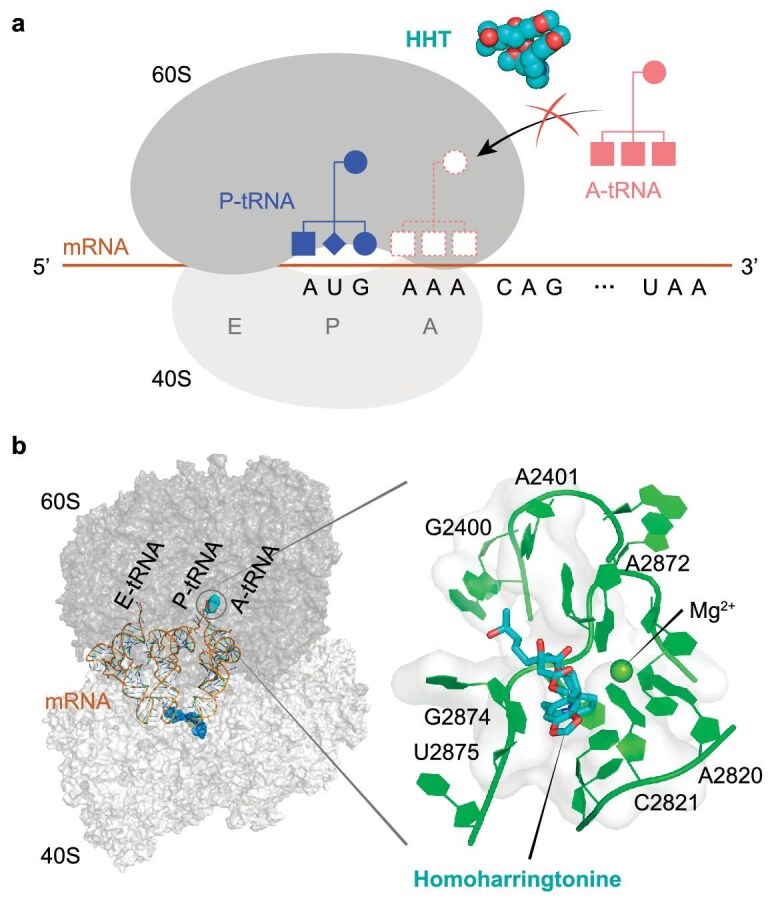
Mechanism of HHT blockage of translation elongation, which underlined its anti-coronavirus efficacy. (a) HHT prevented the incoming aminoacyl-tRNA (A-tRNA) from unloading its amino acid cargo (P-tRNA) to extend the peptide by one amino acid. (b) Structural details of the docking of HHT at the A site of the ribosome, resulting in the inhibition of translation elongation, as shown in (a). The drawing was based on the structural data (PDB code: 4U4Q) in [23].

More specifically, HHT is known to act on peptide elongation (see Fig. 1a). As HHT does not affect translation initiation but is highly efficient at blocking elongation, it is sometimes described as an agent against ‘initial elongation’ [22,23]. Based on the structural data of Garreau de Loubresse *et al*. [23], we modeled the molecular mechanism. The model in Fig. 1b shows graphically how HHT competed with the amino acid side chains of aminoacyl-tRNAs for binding to the A-site cleft of the ribosome. Thanks to the highly specific mechanism of viral repression, we expected HHT to work at a very low dose.

### The *in vitro* efficacy of HHT against coronaviruses (including SARS-CoV-2)

**Figure 2.**
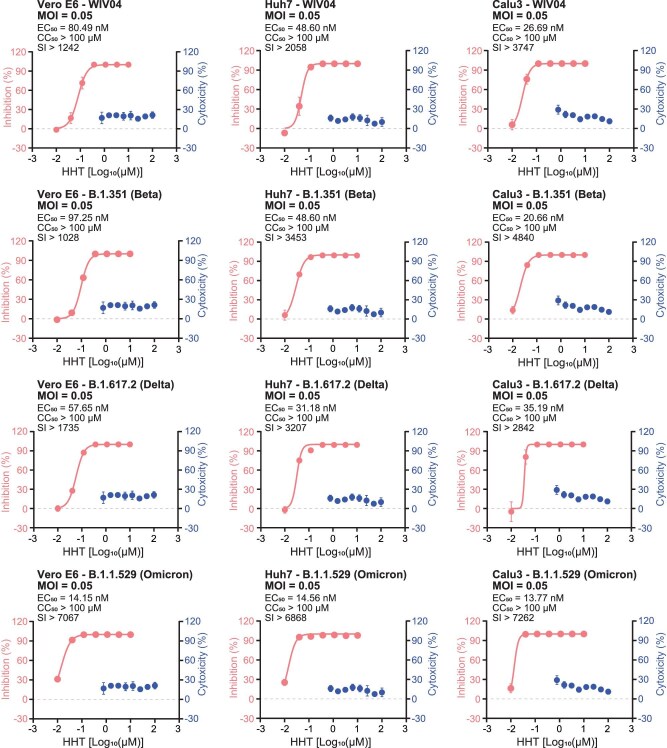
HHT strongly inhibited different strains of SARS-CoV-2 *in vitro*. CC_50_ value of HHT in three human cell lines (Vero E6, Huh7 and Calu3) and EC_50_ value of HHT against different strains of SARS-CoV-2 (wild-type, Beta, Delta and Omicron) were given for these cells. The selectivity index (SI) in each combination was then determined.

After the preliminary investigation in 2020 [2], we systemically studied the antiviral efficacy of HHT against several SARS-CoV-2 variants of concern (VOCs) in three cell models (Vero E6, Huh7 and Calu3). In general, the CC_50_ value of HHT against human cells exceeded the highest tested concentration (100 μM) and the EC_50_ value against any SARS-CoV-2 strains was <100 nM, yielding a substantial SI of >1000 under all test conditions (Fig. 2). When compared with molnupiravir under similar conditions, the efficacy of HHT was much higher than that of molnupiravir in terms of lower EC50, similar CC50 and higher SI (Table 1 and File S1). More importantly, the EC_50_ value against the Omicron strain (B.1.1.529) was only ∼10 nM, which is much lower than those of other antiviral drugs, including PF-07321332 (EC_50_ = 0.07–0.12 μM) [24,25], remdesivir (EC_50_ = 0.05–0.77 μM) [25,26] and molnupiravir (EC_50_ = 0.22–0.4 μM) [27,28].

**Table 1.** Comparison of *in vitro* and *in vivo* anti-SARS-CoV-2 efficacy between HHT and other approved drugs.

*In vitro*												
	Vero E6 infected by WIV04			Vero E6 infected by Beta strain			Vero E6 infected by Delta strain			Calu3 infected by Delta strain		
	EC_50_ (nM)	CC_50_ (μM)	SI	EC_50_ (nM)	CC_50_ (μM)	SI	EC_50_ (nM)	CC_50_ (μM)	SI	EC_50_ (nM)	CC_50_ (μM)	SI
Molnupiravir	340	>200	>588	410	>200	>487.8	440	>200	>454.5	230	>200	>869.6
HHT	80.49	>100	>1242	97.25	>100	>1028	57.65	>100	>1735	35.19	>100	>2842
*In vivo*												
Remdesivir (Gilead)	In a rhesus macaque model, on 7 dpi (days post-infection), virus clearance achieved in 10/36 samples in the remdesivir-treated group, whereas it was 3/36 in the placebo group [36].											
Molnupiravir (Merck)	In an immunodeficient mice implanted with human lung tissue (LoM) model; if molnupiravir treatment started at 24 hours after SARS-CoV-2 exposure, virus clearance achieved in 4/8 samples after 2 days of treatment; if treatment started at 48 hours, virus clearance achieved in 0/8 samples [37].											
Nirmatrelvir (Pfizer)	In a mouse-adapted SARS-CoV-2 model, on 4 dpi, virus clearance achieved in 2/11 mice in the nirmatrelvir-treated group, whereas all mice in the placebo group had robust infection [24].											
HHT (MinSheng)	In a mouse-adapted SARS-CoV-2 model, on 4 dpi, virus clearance achieved in 4/7 mice if treated with HHT by IP, in 6/6 if by IN, whereas all mice in the placebo group had robust infection.											

Furthermore, since the SARS of 2003, many efforts have been made to identify drugs that are capable of repressing coronaviral replication and multiple drugs were reported [29–32,33]. Interestingly, we noticed that HHT (and only HHT) appeared in almost all assays. Five coronaviruses (including murine coronavirus mouse hepatitis virus, bovine coronavirus strain L9 (BCoV-L9), human enteric coronavirus strain 4408 (HECoV-4408), porcine epidemic diarrhea virus (PEDV) and middle East respiratory syndrome coronavirus (MERS-CoV)) had been documented as being repressed by HHT before 2020 [29–31].

**Figure 3.**
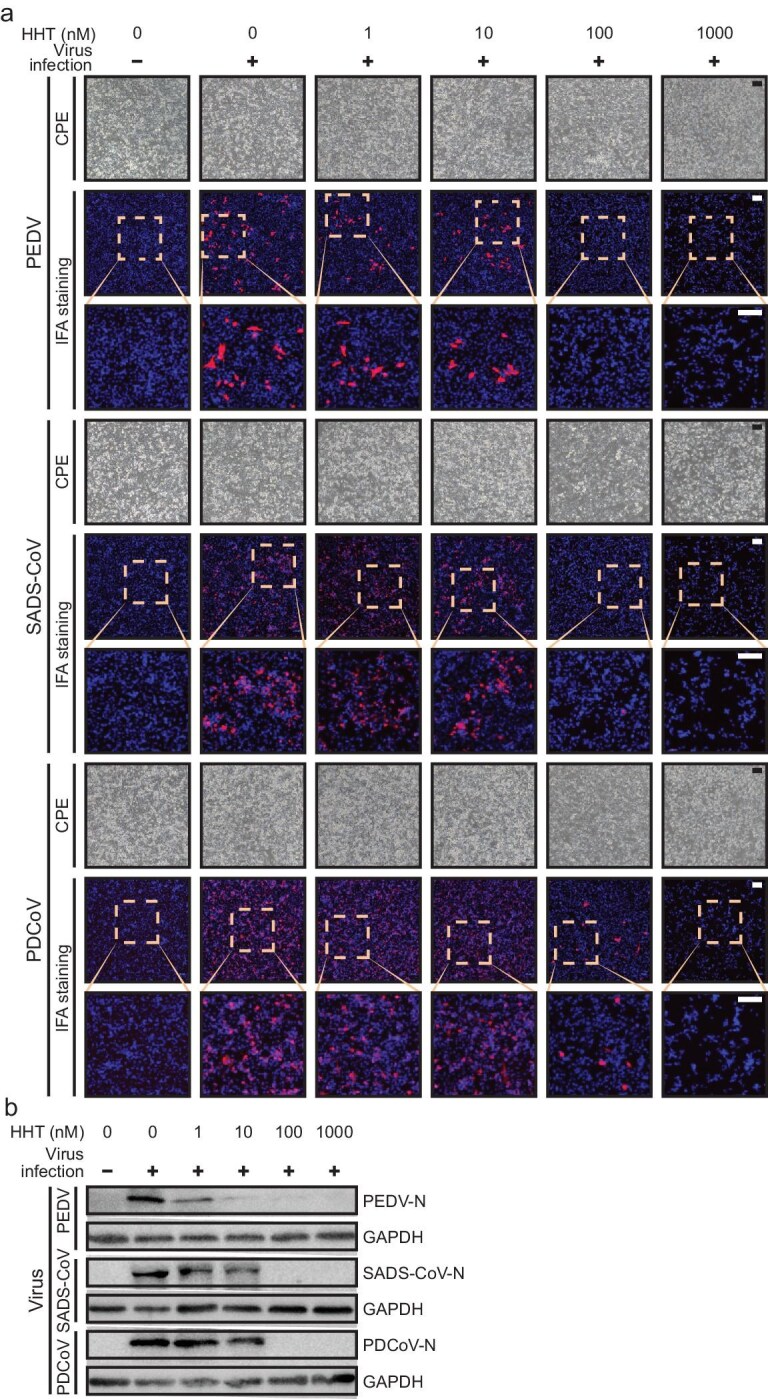
HHT inhibited three different porcine coronaviruses *in vitro* at comparable doses (<100 nM) as against SARS-CoV-2. The IPI-FX cells (porcine intestinal epithelial cells) were treated with various concentrations of HHT (0–1000 nM) or the control normal DMEM for 1 hour, followed by infection with PEDV or SADS-CoV at an MOI of 0.1 or PDCoV at an MOI of 0.01. After 1 hour, the cells were retreated with HHT or DMEM as a control. (a) At 24 hours post-inoculation, the cytopathic effect (CPE) caused by the viruses was observed under microscopy (top row of each panel). Viral antigen was measured by using an indirect immunofluorescence assay (middle and lower rows with zoom-ins shown). (b) Meanwhile, cell lysates were prepared and the expressions of PEDV-N, SADS-CoV-N and PDCoV-N were examined, respectively, using Western blot. Anti-GAPDH was used to represent host proteins.

Because these reports were spread among laboratories that used various dosages, the HHT effect might have been due to diverse factors. To test the hypothesis of a common mechanism, we used PEDV from the previous list [30] and two additional porcine coronaviruses—swine acute diarrhea syndrome coronavirus (SADS-CoV) and porcine deltacoronavirus (PDCoV)—in the same experimental setting. Our goal is to find out whether the dose–response among coronaviruses is indeed similarly low. Figure 3 shows that the IC_50_ was generally ∼100 nM and the eradication was achieved at <1 μM. We noted that these were the same range of values as those reported in the literature [29–33]. In summary, six different coronaviruses, including multiple variant strains of SARS-CoV-2 VOCs (wild-type, Beta, Delta and Omicron), were shown to be repressed by HHT at a comparable concentration. HHT is thus able to act against all the coronaviruses that were tested, likely targeting the shared feature of unusual protein translation.

### The efficacy of HHT in suppressing SARS-CoV-2 in mouse models

We now present the data on HHT *in vivo*. The experiments were performed in both P3 and P4 laboratories, which use different viral strains—one being adapted to mice [34] and the other being a common SARS-CoV-2 strain that infects transgenic mice that carry the human ACE-2 receptor [35].

**Figure 4.**
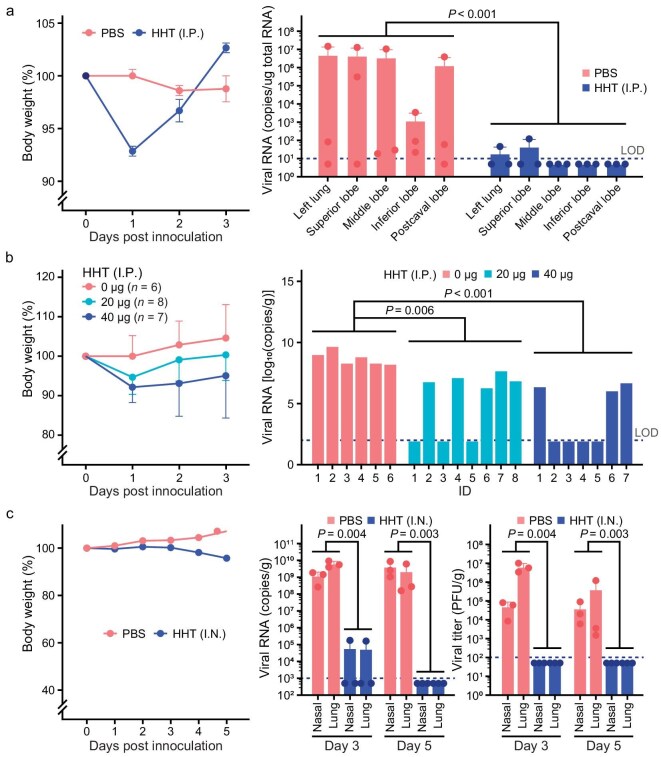
HHT represses SARS-CoV-2 effectively in mouse model experiments, conducted in two facilities. IP (intraperitoneal) injection or IN (intranasal) dripping was administered. IN was adopted later in clinical trials, as it is more efficient at delivery. (a) IP injection of HHT significantly repressed SARS-CoV-2 (strain 107) in hACE2-mice. Left: body weight change during treatment with HHT or PBS (vehicle control). By the one-tailed paired *t*-test, neither was significant (*P* > 0.05). Right: viral load in different parts of lung tissues 3 days after virus inoculation (non-parametric test, *P* < 0.001 between the PBS group and the HHT group). Error bar represents mean with SEM. (b and c) Confirmations of the results of (a) by using a different viral strain (a mouse-adapted strain) in BALB/c mice. In (b), IP injection of HHT is repeated with two different doses. In (c), IN dripping of HHT is found to be more effective at repressing SARS-CoV-2 replication. After virus inoculation, each mouse was treated by PBS or HHT (40 μg/mouse daily). Left: body weight change during treatment; viral load (middle) and viral titer (right) were measured at both the nasal turbinate and the lung tissue 3 or 5 days after virus inoculation (non-parametric test was performed 3 or 5 days after virus inoculation). Error bar represents mean with SEM.

In the initial experiments, we administered the drug at the theoretical concentration of 5 μM on the first day and 2.5 μM on each subsequent day, or ∼80 and ∼40 μg per mouse per day (see ‘Methods’). Lung tissues were collected on Day 3 post-inoculation and divided into five parts (left lung, superior lobe, middle lobe, inferior lobe and postcaval lobe) for each lung. As shown in Fig. 4a, HHT via IP administration appeared to be tolerable for mice and highly effective against SARS-CoV-2 in 3 days of treatment. The viral load was detectable in only one case, in which the left lung and superior lobe showed a residual viral load.

We then administered the drug via IP—either 40 or 20 μg per mouse per day (Fig. 4b). PBS was used as the vehicle control and each group contained six to eight mice. Again, the drug was well tolerated and the antiviral efficacy was high. In the 20-μg group, the viral load was not detectable in three out of eight mice and, in the 40-μg group, the viral clearance rate was four out of seven. In contrast, the viral load in all mice in the control group was very high. Hence, viral eradication was achievable *in vivo*.

Since the virus is concentrated in the URT in the few days post-infection, we further tested the feasibility of eradicating the virus via local (intranasal (IN)) administration of HHT. The drug was delivered by nasal dripping at a dose of 40 μg per mouse per day (Fig. 4c). Most significantly, on Day 3 post-inoculation, virus RNA was detectable in only one mouse and the viral titer was lower than the limit of detection (LOD) in all mice. In comparison with other drugs (including remdesivir, molnupiravir and PF-07321332), we can see the outstanding efficacy of HHT, both *in vitro* and *in vivo*, against SARS-CoV-2 (Table 1) [24,36,37].

### Clinical trials of HHT

Given the mouse model results, we conducted several small-scale clinical trials on COVID-19 patients. Local administration of a low dose of HHT in the URT was adopted to avoid the side effects that are associated with systemic applications.

#### Safety of HHT administration by nebulization

We first addressed the safety of HHT nebulization on non-COVID-19 subjects. In treating leukemia, the standard dosage is 1.25 mg/m^2^ of body area twice daily (∼4.2 mg/day for a patient with 60 kg of body weight) for 14 consecutive days of a 28-day cycle [19]. In this clinical trial, four lung cancer patients (without COVID-19) were recruited. Although the primary goal was cancer treatment, extensive data were collected on the possible adverse effects of nebulizing HHT. In this trial, the therapeutic regimen in a 3-week cycle was as follows: 0.5 mg of HHT per inhalation, twice a day on Days 1–5 of the first week, and 2 days of treatment each week in the next 2 weeks (see details of this clinical trial in https://www.chictr.org.cn/index.aspx through accession number ChiCTR2100049182). The results had been reported in Ma *et al.* (2022). Overall, HHT appeared to be beneficial without any detectable adverse effects after the treatment cycle [38]. Follow-up tests in the ensuing 6 months also did not report adverse effects associated with the treatment. In File S2, we present additional nebulization experiments of HHT on dogs at a concentration of up to eight times the dosage used on humans. Detailed measurements (see File S2) also showed no adverse effects at such a high concentration.

#### Local suppression of SARS-CoV-2 in lung cancer patients with COVID-19

To test the clinical benefit of the local suppression of SARS-CoV-2, we first carried out a preliminary trial by using alcohol, which was reported to reduce oropharyngeal viral load efficiently [2,39]. For this preliminary trial, 28 volunteers with COVID-19 were recruited. Patients were advised to clear the virus in the oral cavity by gargling with alcohol (38%–42% in liquor) for 60–90 seconds [2]. The outcomes are reported in File S3. Briefly, most patients could clear the virus with one single mouthwash as monitored by using the antigen test (Fig. S3A). In cases of unusually heavy viral load, as many as three consecutive washes were needed (Fig. S3B). For about half of them, the virus did not come back after the clearance. In the remaining cases, the antigen test became faintly positive 2 hours after the clearance and reached approximately half the pretreatment strength 6 hours later (Fig. S3C). In summary, alcohol, by merely denaturing mature virions, may alleviate the viral burden on the immune system, which could help a subset of patients. Nevertheless, it is clear that local treatment needs to disrupt viral production to be broadly effective. HHT may serve that purpose.

In November and December of 2022, at the height of the epidemic in China, COVID-19 patients with primary lung cancer or lung metastasis were at great risk. A treatment regime that could halt cancer cell proliferation while reducing the viral load in the respiratory tract could be beneficial. With this main purpose, we initiated a clinical trial to study the safety and efficacy of HHT nebulization in lung cancer patients with COVID-19. As an approved cancer drug with outstanding anti-SARS-CoV-2 efficacy, HHT was approved by the IRB of Clifford Hospital in this trial. In addition, HHT was administrated by nebulization in order to treat cancer cells in the lung tissue. All 26 patients were subjected to two sessions of nebulization treatment each day—one in the morning and the second one 6 hours later for 4 days (see ‘Methods’). The HHT dose was 0.5 mg in each session and swab sampling was performed before each session. Basic information on these patients is included in Table 2.

**Table 2.** Demographic and clinical characteristics of the enrolled cancer patients with COVID-19.

Total number	26
Sex	
Male	17
Female	9
Age, years (median, range)	48 (27–85)
Vaccination status (number (%))	
Unvaccinated	11 (42.3)
Standard course	3 (11.5)
Boosted course	7 (26.9)
Unknown	5 (19.2)
Symptoms	
Fever	19
Fatigue	15
Coughing	16
Expectoration	14
Muscular soreness	5
Pharyngalgia	5
Shortness of breath	6

**Figure 5.**
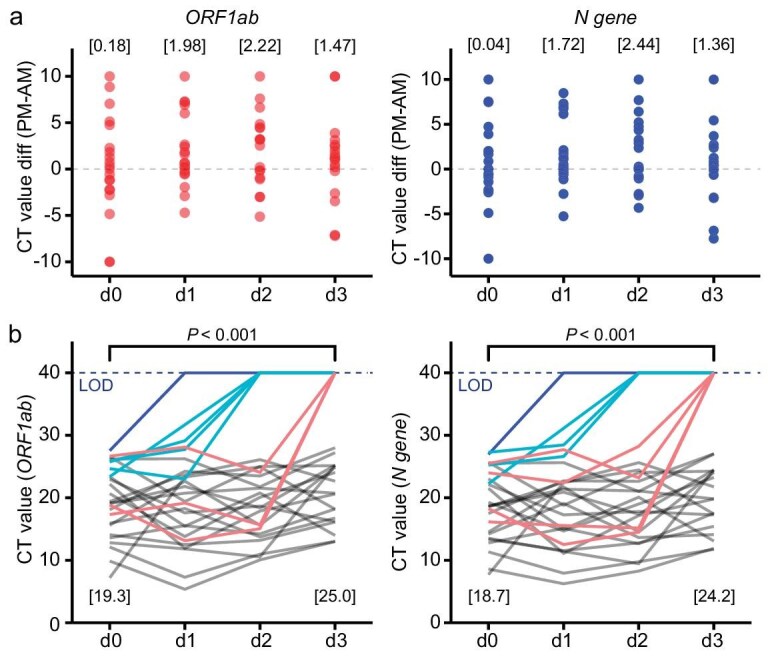
Assessment of the safety and efficacy of HHT nebulization against SARS-CoV-2 in 26 cancer patients. (a) Short-term effect. Each patient was treated in two daily sessions (morning and evening, 6 hours apart) of HHT nebulization. Nasopharynx specimens were taken before each session and their RT-PCR CT values for *N* gene and *ORF1ab* gene were determined. Dots represent the differences in CT values between evening and morning specimens from the same patient. Numbers on the top represent the average CT increases between sessions. On average, the viral load was reduced by two-thirds in 6 hours after treatment (see main text). (b) Longer-term (days) effect. CT values of specimens that were collected during the course of treatment. Numbers on the top represent the median CT values of specimens from these patients before (d0) and after (d3) the treatment (one-sided paired *t*-test, *P*-value was calculated between the CT value at d0 and d3). Dashed lines represent the limit of detection (LOD, CT Value = 40) in this study.

Figure 5a presents the differences between the CT measurements on the same day. They manifested the effect of HHT on the viral load, measured 6 hours apart between the two sessions. While there was no measurable difference in the first day of treatment (d0), the effect was clear in the following 3 days (d1–d3). The average viral load decreased by >70% (+1.98 in Ct, being 25.3%) 6 hours after the morning session.

Over the 4-day course, the median CT value of ORF1ab increased from 19.3 to 25.0 and the N gene CT values increased from 18.7 to 24.2 (Fig. 5b). In aggregate, the CT values increased by ∼5.5 in 3 days and eight patients turned negative in this period (1 patient on the first day, 4 patients on the second day, and 3 patients on the third day). Although the effect was weaker than the extrapolation from the local effect shown in Fig. 5a, the rate of viral reduction that is shown in Fig. 5b was faster than that reported for untreated cancer patients [40]. In summary, HHT nebulization could efficiently halt viral production in cancer patients but viral eradication was not quick enough, likely due to their immuno-compromised status. As it is necessary to deliver HHT to the lungs of these patients, the treatment used was nebulization. A better option for targeting the URT for viral suppression was used in the next section.

#### Suppression of SARS-CoV-2 in newly infected patients without prior health conditions

The results presented above offer three lessons for suppressing the resurgence of epidemics, which would require clearing the virus rapidly in each new infection on a large scale. First, the treatment should start as soon after the infection as possible—preferably within 48 hours while the virus is still concentrated in the URT. Second, patients with uncompromised immunity should be more likely to receive the desired benefits. Third, as the targeted area is the URT, delivery by nasal spray in liquid form (akin to nasal dripping, as shown in Fig. 4c) would be far more effective. By using nebulization, the droplets that are in aerosol form and generally <5 μm in size would travel down to the lungs and, if not absorbed in the alveoli, would be exhaled. Only a small fraction (perhaps <10%) would be absorbed in the nasal, pharyngeal and oral cavities [41].

**Figure 6.**
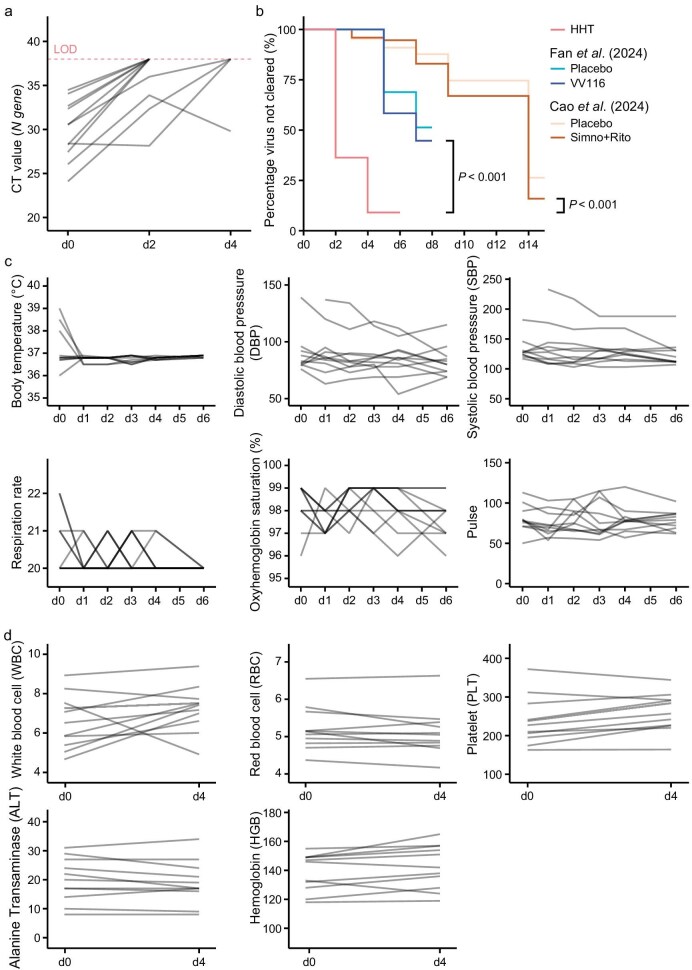
Safety and efficacy of HHT against SARS-CoV-2 by nasal spray. Eleven newly infected individuals who were otherwise normal and healthy participated in this study. HHT was administrated by nasal spray (0.2 mg/day, until viral clearance or after 5 days of treatment) immediately after the diagnosis of SARS-CoV-2 infection (d0). (a) Viral loads were measured during the treatment (d0–d4). Red dashed lines represent the limit of detection (LOD, CT Value = 38). Ten of the 11 patients were cleared of the virus in 2–4 days. Only one patient had a CT value of <30 by d2 and only one did not clear the virus by d4. (b) The efficacy of virus clearance was compared between HHT and other approved drugs (VV116, simnotrelvir plus ritonavir). The efficacy data for VV116 were adopted from [43] and the data for simnotrelvir plus ritonavir were adopted from [42]. Percentage of participants who remained test-positive (*y*-axis) is plotted against the days after treatment (*x*-axis). By using the Log-rank test, the HHT group (red line) is significantly lower than either the VV116 (brown line) (*P* < 0.0001) or the simnotrelvir–ritonavir group (blue line, *P* < 0.0001). (c) Participants’ general physiological conditions were measured during (d0–d4) and 2 days after (d6) the treatment and (d) peripheral hemogram were measured during (d0–d4). No treatment-related adverse events were observed.

With these improvements, we started another investigator-initiated phase I/II clinical trial in Clifford Hospital to study the safety and antiviral efficacy of HHT via nasal spray (IRB approval number IIT2022001). Eleven COVID-19 patients who were otherwise healthy were recruited during the wavelet of infections in May 2023. In this trial, HHT was administrated by using a nasal spray (0.2 mg/day, until viral clearance or for 5 days at most) immediately after the diagnosis of SARS-CoV-2 infection (d0). Note the very low dose of HHT that was used in this trial. The outcomes were significant (Fig. 6). Ten of the 11 patients were cleared of the virus in 2–4 days (Fig. 6a).

Given the nature of the epidemic from late 2022 to early 2023, the control data have to be obtained from the general population who were surveyed at the same time and in the same area. The most suitable comparisons are now described [42,43]. Cao *et al*. [42] reported the efficacy of oral simnotrelvir (targeting 3CLpro of SARS-CoV-2, similarly to nirmatrelvir) for adult patients with mild-to-moderate COVID-19. The trial was performed from August to December 2022 and enrolled 1208 patients at 35 sites in China. In this trial, fewer than half of the patients with SARS-CoV-2 infection turned negative within 9 days after the initiation of treatment. The simnotrelvir/ritonavir and placebo groups show little difference. Importantly, both groups took far longer to clear the virus than those in the HHT trial (Fig. 6b). In another trial, Fan *et al*. [43] reported the efficacy of oral VV116 (targeting RdRp of SARS-CoV-2, similarly to remdesivir) in patients with mild-to-moderate COVID-19 in China over a similar time span, between October 2022 and January 2023. Again, the efficacy at clearing SARS-CoV-2 was worse than that of HHT, as shown in Fig. 6b. Despite the small sample size in the HHT trial, the substantial differences in the time to clearance between our study (2–4 days) and the two contemporary trials in China (7–9 days) were highly significant when using the Log-rank test (Fig. 6b).

Taken together, HHT appeared to be more effective than simnotrelvir/ritonavir or VV116 at accelerating SARS-CoV-2 clearance in newly infected patients. Meanwhile, the general physiological conditions (Fig. 6c) and peripheral hemograms (Fig. 6d) of participants were monitored and no treatment-related adverse events were observed. In summary, a very low dose of HHT (0.2 mg/day) that was administered by nasal spray showed superior safety and antiviral efficacy in COVID-19 patients without other health conditions. HHT thus meets the criteria for the population-level suppression of epidemics.

## DISCUSSION

Given the current state of human societies (population densities, social interactions, human movements, etc.), epidemics that are caused by coronaviruses should be considered a constant threat. It has been reported that coronavirus transmission may occur frequently by asymptomatic individuals, making them particularly dangerous [44,45]. We should note again that COVID-19 has not yet vanished; hence, its resurgence, albeit seemingly remote, cannot be ruled out [46].

The development of an HHT scheme based on the proposal of 2020 [1,2] includes the elucidation of the inhibitory and structural mechanism of protein elongation, the extensive evaluation of its antiviral efficacy against multiple SARS-CoV-2 strains (including wild-type, Beta, Delta and Omicron), its broad anti-coronavirus activity and the strong anti-SARS-CoV-2 performance in animal models in multiple facilities. In addition, the small-scale clinical trial in this study has demonstrated the potential of HHT as a first-line defense in future coronavirus epidemics.

From the public health stand point, the goal is to develop a drug that can suppress the virus in the URT immediately after infection. The application should be feasible on a large scale economically (such as a nasal spray). We believe that the efficacy of HHT in suppressing future coronavirus epidemics is promising. For that reason, strategies that are based on HHT can be developed long before any actual epidemics. They may include (i) safety data on HHT administration via nasal spray obtained by using systematic phase I trials and (ii) systematic efficacy data on other coronaviruses in animal models such as those shown in Fig. 4.

We now briefly review the drug developments and therapeutic applications that occurred during COVID-19. Between 2020 and 2024, hundreds to thousands of potential drugs were invested in or prepared for therapeutic applications. They fall into three major categories.

First, some anti-spike monoclonal antibodies (such as bebtelovimab, sotrovimab and regdanvimab) have been approved to treat outpatients with mild-to-moderate COVID-19. Because anti-spike monoclonal antibodies are costly and can be easily evaded by SARS-CoV-2 variants (Omicron, for instance) [47], they could not suppress epidemics at the population level.

Second, several previously approved immunomodulatory drugs (such as dexamethasone, cytokine antagonist tocilizumab and Janus kinase inhibitor baricitinib) have also been approved to treat hospitalized patients with moderate-to-severe COVID-19. The benefit of immunomodulatory drugs depends largely on the timing of drug administration, dosage and disease severity [48]. While these drugs could potentially be curative, they may not be effective at suppressing viral spread.

The third category comprises many small-molecule antiviral drugs. They include RdRp inhibitors (such as remdesivir and molnupiravir) and M^pro^ inhibitors (such as nirmatrelvir–ritonavir (i.e. PAXLOVID) and ensitrelvir). These four drugs are perhaps the most widely used medications for COVID-19 thanks to promising performance in the early trials. Beyond those, many other small-molecule antiviral drugs did not show beneficial effects in early trials and are not commented upon here.

Due to the compressed time frame during the pandemic, rigorous evaluations of the efficacies of previously promising drugs are possible only in hindsight. For remdesivir, the WHO Solidarity trial, which enrolled 11 330 adults, did not show positive effects on hospitalized patients [11]. (The trial also tested other types of drugs, including hydroxychloroquine, lopinavir and interferon regimens with little positive outcome.) Similarly, in the PANORAMIC trial, which enrolled 26 411 participants, molnupiravir did not reduce the frequency of COVID-19-associated hospitalizations or death among high-risk vaccinated adults in the community [12].

As for PAXLOVID, which may be the most promising of the drugs that were developed during COVID-19, the time to sustained alleviation of all signs and symptoms of COVID-19 did not differ significantly between participants who received the treatment and those who received placebo [13]. In addition, post-exposure prophylaxis with PAXLOVID for 5 or 10 days did not significantly reduce the risk of symptomatic SARS-CoV-2 infection [49].

In summary, effective treatment strategies that can be inexpensively and conveniently applied are still highly desirable in preparation for future outbreaks of pathogenic coronaviruses [48]. The HHT scheme may be a possible candidate. We note that the scheme was first published in April, and then in October, 2020 [1,2]. However, it was not possible to conduct a clinical trial for want of COVID-19 patients until the large waves of COVID-19 in China in late 2022 and early 2023. Prior to that, individual scientists were interested in collaboration (see ‘Acknowledgements’) but cross-nation mechanisms for such collaborations were limited. These mechanisms should be developed before the next pandemic.

## METHODS

### Facility, ethics and biosafety statement

The main *in vitro* experiments on viral infection were performed in a Biosafety Level 3 (BSL-3) laboratory in Wuhan Institute of Virology (WIV), Chinese Academy of Sciences. Authenticated viruses of four SARS-CoV-2 strains—WIV04 [50], B.1.351 (NPRC2.062100001), B.1.617.2 (IVCAS-6.7585) and B.1.1.529 (IVCAS-6.7600)—were obtained from the National Virus Resource Center (Wuhan, China). The amplification of the virus and determination of the median tissue culture infective dose (TCID50/mL) were performed in Vero E6 cells, also in WIV, as stated above.

The *in vivo* experiments with infectious SARS-CoV-2 were performed in the Biosafety Level 4 and animal Biosafety Level 4 facilities in the Harbin Veterinary Research Institute (HVRI) of the Chinese Academy of Agricultural Sciences (CAAS), approved by the Ministry of Agriculture and Rural Affairs of China.

Earlier *in vivo* antiviral studies were carried out under BSL-3 conditions at the Key Laboratory of Animal Models and Human Disease Mechanisms of the Chinese Academy of Sciences (CAS), Kunming Institute of Zoology (KIZ). The animal studies were carried out in strict accordance with the recommendations in the Guide for the Care and Use of Laboratory Animals of the Ministry of Science and Technology of the People's Republic of China. The protocols were approved by the Committee on the Ethics of Animal Experiments of the WIV of CAS, HVRI of CAAS or Institutional Committee for Animal Care and Biosafety at KIZ, CAS, respectively. All animals used in this study were chosen randomly.

### Cells and viruses

At WIV, African green monkey kidney clone E6 (Vero E6; American Type Culture Collection (ATCC), no. 1586) cells were cultured at 37°C with 5% CO_2_ in minimum Eagle's medium (Gibco, Grand Island, NY, USA) supplemented with 10% fetal bovine serum (FBS, Gibco). Human hepatocarcinoma cell line-Huh7 (National Virus Resource Center, IVCAS 9.005) and human lung epithelial cells—Calu-3 (HTB-55, ATCC)—were maintained in Dulbecco's modified Eagle's medium (DMEM) supplemented with 10% FBS.

In HVRI, Vero E6 cells were maintained in DMEM containing 10% FBS and antibiotics and incubated at 37°C with 5% CO_2_. Mouse-adapted SARS-CoV-2/HRB26/human/2020/CHN (HRB26M, GISAID access no. EPI_ISL_459 910) was obtained by serially passaging the HRB26 virus in 4- to 6-week-old female mice until passage 14 and was propagated in Vero E6 cells. Infectious virus titers were determined by using a plaque forming unit (PFU) assay in Vero E6 cells.

In KIZ, SARS-CoV-2 (strain 107) was provided by Guangdong Provincial Center for Disease Control and Prevention (Guangzhou, China). This virus was propagated and titrated on Vero E6 cells (ATCC, no. 1586), which were cultured in DMEM (Gibco) with 4.5 mM of L-glutamine (GE Life Sciences) supplemented with 10% FBS (Hyclone) and 1% penicillin–streptavidin (Gibco). Cells were cultured at 37°C in a humidified 5% CO_2_ atmosphere. Mycoplasma testing was performed at regular intervals and no mycoplasma contamination was detected.

### Chemical compounds

HHT, purchased from APExBIO (Catalog No. N1504), was used in all *in vitro* and animal studies. In the clinical trial of ChiCTR2100049182, HHT injection (1 mg/mL) was provided by MinSheng Pharmaceuticals Co., Ltd.

### *In vitro* antiviral studies of HHT

Homoharringtonine (APExBIO, N1504) was dissolved in dimethyl sulfoxide and diluted to the working concentration with cell culture medium. To test the cytotoxicity of HHT, a series of diluted concentrations (0.78–100 μM) were incubated with Vero E6, Huh7 or Calu-3 cells in a 96-well plate (2 × 10^4^ cells/well) for 24 hours, following cell viability assessment by using a cell-count kit-8 (Beyotime, Shanghai, China, #C0039) according to the manufacturer's instructions.

To test the antiviral effect of HHT, cells were incubated with HHT following infection with SARS-CoV-2 (MOI = 0.05) for 24 hours (Vero E6 and Huh7) or 48 hours (Calu-3), respectively. The antiviral effect was measured by using a previously reported approaches quantification of the cell supernatant to assess the progeny virus yield [26]. EIDD-1931 (the active form of molnupiravir) was used as the positive control in this assay. This part was conducted in WIV.

### *In vivo* antiviral studies of HHT

The mice for this study, which were 8- to 10-week-old BALB/c mice (half male and half female), were obtained from Beijing Charles River Labs (Beijing, China). Mice were lightly anesthetized with CO_2_ and intranasally inoculated with 50-μL dilutions of SARS-CoV-2. Body weights and clinical symptoms were monitored daily. In the high-dose (40 μg) group, seven mice were treated by intraperitoneal (IP) injection with a loading dose of 40 μg/mouse/day of HHT. In the low-dose (20 μg) group, eight mice were treated by IP injection with a loading dose of 20 μg/mouse/day of HHT. As a control, six mice were administered vehicle solution (PBS) daily. The mice from each group were euthanized on Day 3 post-inoculation. The lungs were collected for virus detection by using qPCR and PFU assay [34,35]. In an alternative mode, after the inoculation of SARS-CoV-2, groups of six mice were treated intranasally (IN) with HHT (40 μg/mouse daily) or PBS (as vehicle control). Three mice from each group were euthanized on Days 3 and 5 post-inoculation. The nasal turbinates and lungs were collected for virus detection by using qPCR and PFU assay [34,35]. This part was conducted in HVRI.

Additional experiments that were carried out in KIZ were part of the multi-laboratory design. The angiotensin-converting enzyme 2 (ACE2) humanized mice (hACE2 mice) aged 8–10 weeks were generated from Guangzhou Institute of Biomedicine and Health, CAS [35]. Mice were anesthetized with isoflurane (RWD Life Science, Shenzhen) and intranasally infected with 2 × 10^6^ TCID_50_ of SARS-CoV-2 (strain 107) in 30 μl of DMEM. Body weights and clinical symptoms were monitored daily. The treated group of three mice were treated intraperitoneally with a loading dose of 80 μg/mouse HHT, followed by a daily maintenance dose of 40 μg/mouse. As a control, mice (*n* = 3) were administered vehicle solution (PBS) daily. Lung tissues were collected on Day 3 post-inoculation. RNA extraction and viral RNA quantification were performed according to a previous study [51].

### Evaluation of the antiviral efficacy of HHT against other coronaviruses

IPI-FX cells (a subcloned line of homogeneous cells that are derived from IPI-2I cells (porcine intestinal epithelial cells)) were obtained from Wen’s Foodstuffs Group Co., Ltd (Guangdong, China) [52]. Cells were cultured in DMEM (Hyclone, USA) supplemented with 100 U/mL of penicillin, 100 U/mL of streptomycin and 10% FBS (BOVOGEN, Australia). The maintenance medium for PEDV, SADS-CoV or PDCoV propagation was DMEM supplemented with 7.5 μg/mL of trypsin (Gibco, USA) [53].

Confluent IPI-FX cell monolayers, in a 12-well plate, were inoculated with various concentrations of HHT (1–1000 nM) or the control of normal DMEM for 1 hour, followed by infection with PEDV, SADS-CoV or PDCoV at an MOI of 0.1 (PEDV, SADS-CoV) or 0.01 (PDCoV) for 1 hour, and then the viral inoculums were removed and fresh maintenance medium that contained different concentrations of HHT was added. Twenty-four hours later, cells were checked under microscopy to observe the cytopathic effect and then fixed for indirect immunofluorescent assay (IFA). Meanwhile, cell lysates were prepared and the expressions of PEDV-N (nucleocapsid gene), SADS-CoV-N and PDCoV-N were examined, respectively, by using Western blot. GAPDH was also examined as representing the host proteins in each Western blot. Briefly, in the IFA, cells were fixed with 4% paraformaldehyde for 15 minutes and then permeabilized with 0.2% Triton X-100 for 15 minutes at room temperature. After they were blocked with 1% bovine serum albumin (BSA), cells were stained with anti-PEDV (SADS-CoV or PDCoV) N polyclonal antibody (Wen’ s Foodstuffs Group Co., Ltd, China) (1:1000) at 37°C for 1 hour. Cells were then washed with 1 × PBS and incubated with fluorescein isothiocyanate (1:500) or Cy3-labeled goat anti-mouse secondary antibody (KPL, USA) (1:500) at 37°C for 1 hour. After three washes in 1 × PBS, cells were counter-stained with DAPI and observed by using a fluorescence microscope (LEICA DMi8, Germany). In Western blot, whole-cell lysates were prepared with RIPA lysis buffer (Beyotime, China) supplemented with 1% protease inhibitors (Beyotime, China) and boiled with 5 × SDS loading buffer for 10 minutes before the same volume of protein samples were fractionated by using 10% SDS-PAGE electrophoresis and the resolved proteins were transferred onto PVDF membranes (Millipore, USA). After they were blocked with 5% BSA, the membranes were incubated for overnight at 4°C with anti-PEDV-N polyclonal antibody (Wen’s Foodstuffs Group Co., Ltd, China) (1:1000), anti-SADS-CoV-N polyclonal antibody (Prof. Yongchang Cao, China) (1:1000), anti-PDCoV-N polyclonal antibody (Prof. Yongchang Cao, China) (1:1000) or anti-GAPDH antibody (Proteintech Group, Inc., USA) (1:10 000). The blots were subsequently incubated with HRP-conjugated goat anti-mouse IgG (1:5000) (Beijing Ray Antibody Biotech, China) for 1 hour at room temperature. The blots were visualized by using the ECL reagent according to the manufacturer's instructions (Beyotime, China).

### Evaluation the safety and efficacy of HHT nebulization in cancer patients

Patients with primary lung cancer or lung metastasis were enrolled in this study. The ethical statement and study protocols have been described in a previous publication [38]. Briefly, HHT nebulization was administrated to treat the patients’ lung cancer. In each inhalation, 0.5 mg of HHT injection was diluted with 2.5 mL of normal saline and nebulized in ∼10 minutes. The therapeutic regimen for 4 consecutive days was as follows: 0.5 mg of HHT per inhalation, twice a day (one in the morning and the other in the afternoon) in d0–d3. From November to December 2022, 26 patients were infected by SARS-CoV-2 in hospitalization and we measured their viral load in the nasopharynx during the nebulization treatment. In most of the patients, we measured their viral load at the first day of HHT nebulization (d0).

### Evaluation of the safety and efficacy of HHT by nasal spray in COVID-19 patients without other health conditions

Newly infected patients without other medical conditions were enrolled in this study. Briefly, a nasal spray of HHT (0.2 mg of HHT injection diluted with 4.8 mL of normal saline) was applied about five times each day for each patient until viral clearance or for 5 days at most. During the treatment, the viral load in the nasopharynx, general physiological conditions (including body temperature, blood pressure (DBP and SBP), respiration rate, oxyhemoglobin saturation degree and pulse) and peripheral hemogram (including WBC, RBC, PLT, ALT and HGB) were measured each day.

## Supplementary Material

nwae382_Supplemental_Files
