# Supplementary material for: Homoharringtonine is highly effective against SARS-CoV-2: a potential first-line defense in future coronavirus epidemics
Source: Natl Sci Rev. 2024 Oct 26;12(11):nwae382. doi: 10.1093/nsr/nwae382 (PMC12661574; doi:10.1093/nsr/nwae382)

**The reliability of the *in vitro* assay platform in WIV**

Our team (in Wuhan Inst. of Virology) set up a standard anti-SARS-CoV-2 drug evaluation system immediately after the outbreak of COVID-19, and for the first time reported Remdesivir and chloroquine had in vitro anti-SARS-CoV-2 activity(*1*). When evaluating the in vitro efficacy of HHT, the EC_50_ values of a positive drug EIDD-1931 (the active form of Molnupiravir) against some SARS-CoV-2 strains (WIV04, Beta, and Delta) were determined on Vero E6 and (or) Calu-3 cells. All the tests employed the same virus inoculum (MOI = 0.05) from the same virus stock for each strain, and the seeded cells were within a narrow passage window, and the drug inhibition potency was calculated according to a previously established method(*1*), so the EIDD-1931 (positive control) and HHT shared similar conditions for this assay. As shown in the below figure (**Figure S1**), the EC50 value of EIDD-1931 against Wuhan strain on Vero E6 was 0.34 μM(**Figure S1A**), which was quite close to a reported data (EC50 = 0.30 μM)(*2*). Therefore, our evaluation system for anti-SARS-CoV-2 was stable and convincing.

**Reference**

1. M. Wang *et al.*, Remdesivir and chloroquine effectively inhibit the recently emerged novel coronavirus (2019-nCoV) in vitro. *Cell Res* **30**, 269-271 (2020).

2. T. P. Sheahan *et al.*, An orally bioavailable broad-spectrum antiviral inhibits SARS-CoV-2 in human airway epithelial cell cultures and multiple coronaviruses in mice. *Sci Transl Med* **12**, eabb5883 (2020).

**Figure legends**

**Figure S1**: EIDD-1931 inhibited SARS-CoV-2 replication efficiently in vitro. Under almost the same conditions as for the antiviral activity assay for HHT, EIDD-1931 was tested against WIV04, B.1.351, B.1.617.2 on Vero E6 cells (A, B, C), and against B.1.617.2 on Calu-3 cells (D). The selectivity index (SI) was calculated by CC50 value divided EC50 value.

**Figure S1**


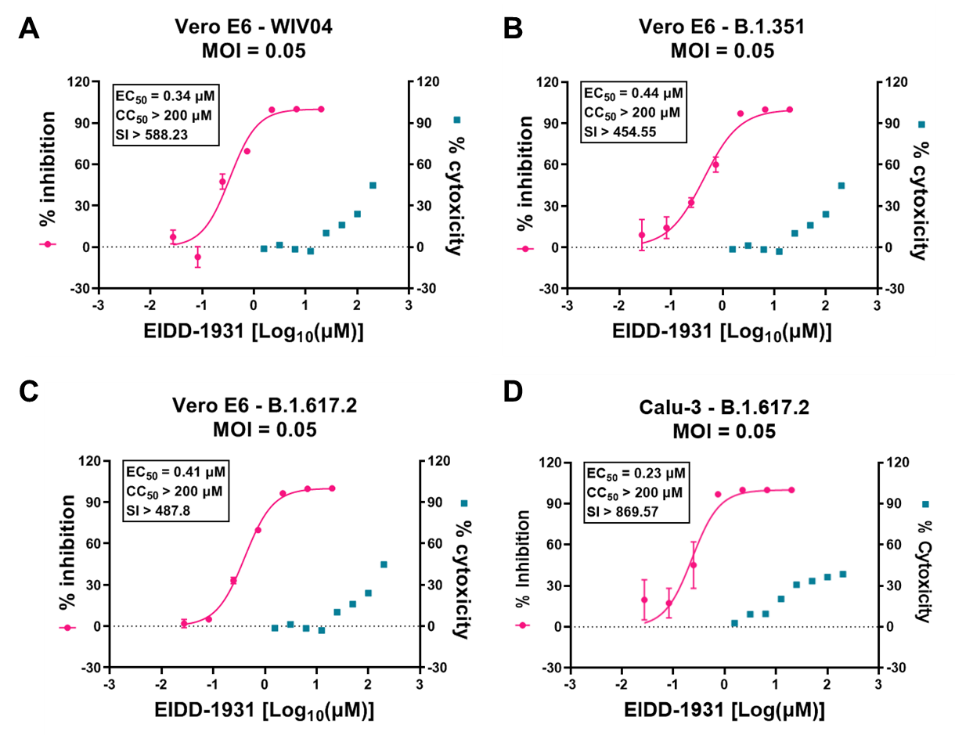

Supplement: nwae382_Supplemental_Files [file nwae382_supplemental_files.zip › HHT-Supplementary file 1.docx]
