# Supplementary material for: Homoharringtonine is highly effective against SARS-CoV-2: a potential first-line defense in future coronavirus epidemics
Source: Natl Sci Rev. 2024 Oct 26;12(11):nwae382. doi: 10.1093/nsr/nwae382 (PMC12661574; doi:10.1093/nsr/nwae382)

**The safety of HHT nebulization in a canine model**

To evaluate the safety of HHT delivered by nebulization in canine, four beagles (female) weighing 10–12 kg were used in this study. The dogs were judged to be in good health based on the results of physical examinations, complete blood cell counts, and serum biochemical analyses. Each dog was fed with an appropriate amount of food and their health status was monitored daily by a dedicated veterinarian.

One dog was assigned to each of the following nebulization dosage: 0.5mg/day, 1.0mg/day, 1.5mg/day, or normal saline alone (control group). Dogs were treated for 7-10 days. Nebulization of HHT was performed in the animal research facility at the University of Kunming Medical University, using a commercially available ultrasonic nebulizer (particle size arounds 5 microns) connected to a polyethylene rebreathing bag. The polyethylene bag was held manually over the muzzle of the dog during treatments (15-20 minutes).

Blood samples were collected daily for complete blood cell counts and serum biochemical analyses. At the end of treatment, the dog received 1.5mg HHT per day was sacrificed and the lung tissue was collected for histopathological study. The other three dogs were adopted.

By the end of the experiment, all dogs appear normal in body weight, blood cell count and blood biochemistry as shown in **Figure S2 (A-C)**. The dog receiving the highest dose was euthanized and the autopsy appears normal as well. In short, dogs could tolerate HHT nebulization at doses much higher than the calculated dosage for treating human patients.

**Figure legends**

**Figure S2**: Evaluation the safety of HHT delivered by nebulization in a canine model. (A) Body weight changes during treatment. No adverse effects were detected (T-test assuming all data displays a normal distribution, p>0.05). (B) complete blood cell count. (C) Biochemistry test. Values at (B) and (C) were standardized by z_i,j_ = (x_i,j_ - μ_j_) / σ_j_ of the normal distribution. Each dot was a sample, taken every day, from an experimental dog. For each test, three treated groups were compared with the control separately using Mann-Whitney U test and Bonferroni correction for multiple comparisons. The four measurements (amylase (AMY) (p=0.008), cholesterol (CHOL) (p=0.008), and glutamic-pyruvic transaminase (ALT) (p=0.008) in the 0.5mg/day group, and CHOL in the 1.5mg/day group (p=0.008)) which were significantly different from the value in control group (Mann-Whitney U test and Bonferroni correction for multiple comparisons) were marked by mocha arrows. Since all values in the four measurements were within the reference range, we concluded that these dosages administrated by nebulization were well-tolerated in dogs.

**Figure S2**


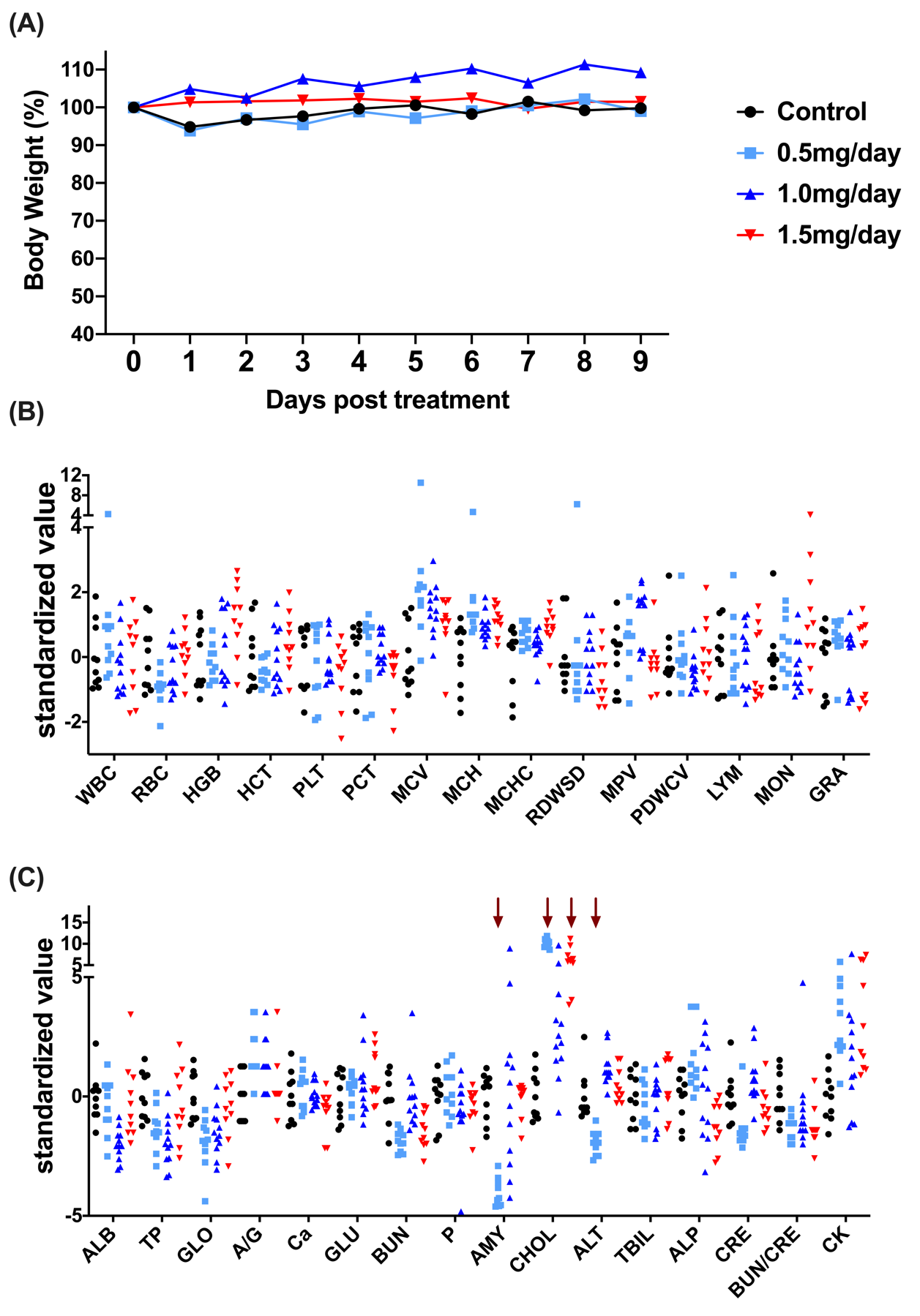

Supplement: nwae382_Supplemental_Files [file nwae382_supplemental_files.zip › HHT-Supplementary file 2.docx]
