# Supplementary material for: Homoharringtonine is highly effective against SARS-CoV-2: a potential first-line defense in future coronavirus epidemics
Source: Natl Sci Rev. 2024 Oct 26;12(11):nwae382. doi: 10.1093/nsr/nwae382 (PMC12661574; doi:10.1093/nsr/nwae382)

**Clearance of SARS-CoV-2 by mouthwash using alcohol or PVP-I**

To test the anti-viral efficacy of mouthwash using alcohol or PVP-I, we recruited volunteers of COVID-19 patients and advised them to clear the virus in the oral cavity by gargling with alcohol (38% - 42% in liquor) or PVP-I solution (1.0% titratable iodine, Hangzhou Minsheng Pharma.). The method of gargling was described in our previous publication(*1*). Briefly, a baseline oropharynx swab specimen was taken for each volunteer before gargling. After the confirmation of SARS-CoV-2 infection using commercially available antigen test kits, the volunteers gargled 3 times using alcohol or PVP-I solution. Subsequently, the volunteers take another oropharynx swab specimen and test the antigen again. Antigen tests were further performed 2 hours, 6 hours, and the next morning after the gargling.

During the wave of infections spread through China in November to December of 2022, more than 30 volunteers in the academic communities in Guangzhou with COVID-19 participated in the self-administered trial using antigen test kits. **Fig. S3A** presented the common observation, achieved with 60-90 seconds of gargling. **Fig. S3B** was a case with an unusually heavy viral load that required 3 consecutive washes. For about half of them, the virus did not come back after the clearance. In the remaining cases, the antigen test became faintly positive 2 hours after the clearance and reached approximately half the pre-treatment strength 6 hours later (**Fig. S3C**). In summary, alcohol, by merely denaturing mature virions, may alleviate the viral burden on the immune system, which could help a subset of patients. Nevertheless, it is clear that local treatment needs to disrupt viral production to be broadly effective.

**Reference**

1. H. J. Wen *et al.*, A proposal for clinical trials of COVID-19 treatment using homo-harringtonine. *Natl Sci Rev* **8**, nwaa257 (2021).

**Figure legends**

**Figure S3**: Local clearance of virus by mouthwash using alcohol or PVP-I. (A) A typical result of the effect of alcohol (38% in liquor) gargling. The virus was undetectable after 90 second wash. (B) A case with an unusually heavy viral load that required 3 consecutive (but shorter) washes to clear. (C) By mouthwash only, the virus may become detectable again 6 hours after clearance.

**Figure S3**


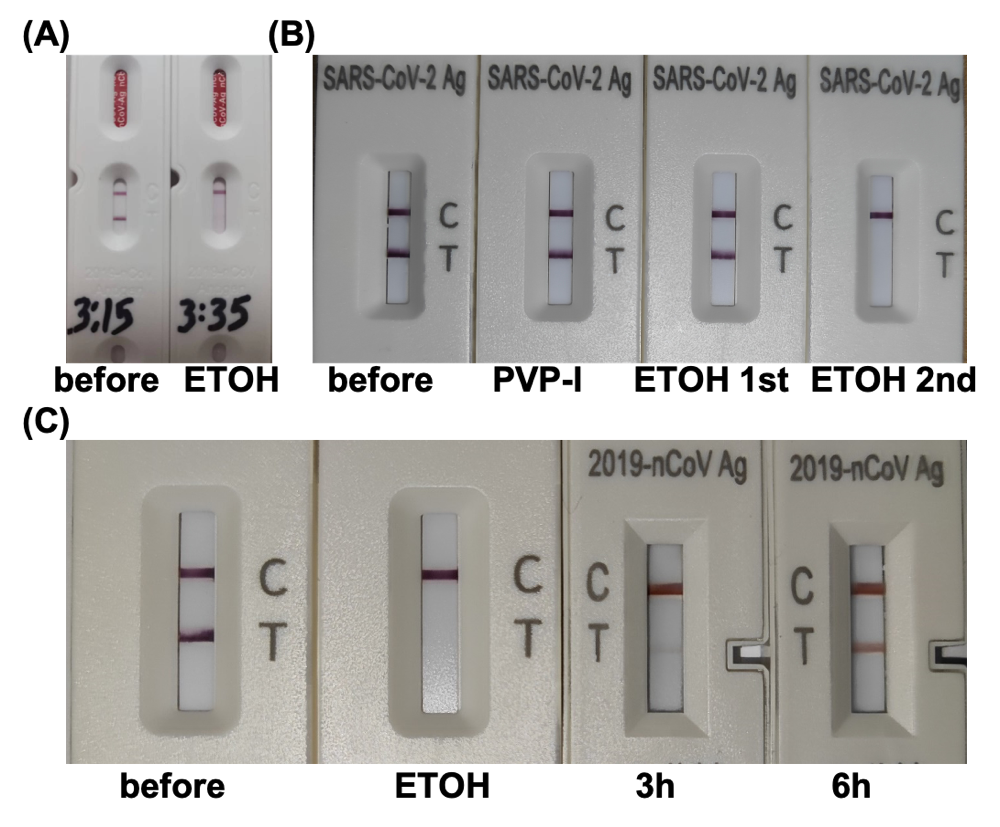

Supplement: nwae382_Supplemental_Files [file nwae382_supplemental_files.zip › HHT-Supplementary file 3.docx]
